# Decreasing CB_1_ receptor signaling in Kupffer cells improves insulin sensitivity in obese mice

**DOI:** 10.1016/j.molmet.2017.08.011

**Published:** 2017-09-01

**Authors:** Tony Jourdan, Sarah M. Nicoloro, Zhou Zhou, Yuefei Shen, Jie Liu, Nathan J. Coffey, Resat Cinar, Grzegorz Godlewski, Bin Gao, Myriam Aouadi, Michael P. Czech, George Kunos

**Affiliations:** 1 Laboratory of Physiologic Studies, National Institute on Alcohol Abuse and Alcoholism (NIAAA), National Institutes of Health (NIH), Bethesda, MD 20852, USA; 2 Laboratory of Liver Diseases, National Institute on Alcohol Abuse and Alcoholism (NIAAA), National Institutes of Health (NIH), Bethesda, MD 20852, USA; 3 Program in Molecular Medicine, University of Massachusetts Medical School, Worcester, MA 01605, USA; 4 Integrated Cardio Metabolic Centre, Department of Medicine, Karolinska Institutet, Huddinge, Sweden

**Keywords:** CB_1_ receptors, Kupffer cells, Insulin resistance, Inflammation, siRNA

## Abstract

**Objective:**

Obesity-induced accumulation of ectopic fat in the liver is thought to contribute to the development of insulin resistance, and increased activity of hepatic CB_1_R has been shown to promote both processes. However, lipid accumulation in liver can be experimentally dissociated from insulin resistance under certain conditions, suggesting the involvement of additional mechanisms. Obesity is also associated with pro-inflammatory changes which, in turn, can promote insulin resistance. Kupffer cells (KCs), the liver's resident macrophages, are the major source of pro-inflammatory cytokines in the liver, such as TNF-α, which has been shown to inhibit insulin signaling in multiple cell types, including hepatocytes. Here, we sought to identify the role of CB_1_R in KCs in obesity-induced hepatic insulin resistance.

**Methods:**

We used intravenously administered β-D-glucan-encapsulated siRNA to knock-down CB_1_R gene expression selectively in KCs.

**Results:**

We demonstrate that a robust knock-down of the expression of *Cnr1*, the gene encoding CB_1_R, results in improved glucose tolerance and insulin sensitivity in diet-induced obese mice, without affecting hepatic lipid content or body weight. Moreover, *Cnr1* knock-down in KCs was associated with a shift from pro-inflammatory M1 to anti-inflammatory M2 cytokine profile and improved insulin signaling as reflected by increased insulin-induced Akt phosphorylation.

**Conclusion:**

These findings suggest that CB_1_R expressed in KCs plays a critical role in obesity-related hepatic insulin resistance via a pro-inflammatory mechanism.

## Highlights

• CB_1_R signaling promotes hepatic insulin resistance by promoting hepatic steatosis and hepatic inflammation.
• CB_1_R knock-down in liver macrophages (Kupffer cells, KCs) improves global insulin resistance and glucose homeostasis.
• CB_1_R expressed in KCs play a critical role in hepatic insulin resistance independent of ectopic fat in the liver or adipose tissue inflammation.

## 1 Introduction

Obesity is a risk factor for developing insulin resistance, which is defined as the inability of cells to respond normally to insulin. A commonly held view is that in a subset of obese, insulin-resistant individuals, β-cell dysfunction ensues, leading to decreased insulin production, poor blood glucose regulation, and ultimately type 2 diabetes (T2D) [1], [2]. The endocannabinoid system (ECS) is comprised of G-protein coupled cannabinoid 1 and 2 receptors (CB_1_/_2_R), their endogenous lipid ligands or endocannabinoids, and synthesizing and degrading enzymes. The discovery of the ECS has triggered an avalanche of experimental studies implicating it in a growing number of physiological/pathological functions [3], [4]. Modulation of ECS activity both in the peripheral and central nervous systems and in various peripheral organs with specific antagonists holds therapeutic promise for a broad range of diseases such as inflammatory disorders or obesity/metabolic syndrome, among others [4]. The mechanisms that link obesity and insulin resistance are the subject of intensive research, with increasing evidence for a major role of inflammation. Specifically, the development of excess, including ectopic, adipose tissue (AT) is strongly associated with chronic inflammation caused by infiltration of activated immune cells and overproduction of pro-inflammatory cytokines. Pro-inflammatory cytokines, such as TNF-α, can block insulin receptor signaling in multiple cell types, including adipocytes and hepatocytes [5], [6]. It is well known that nonalcoholic fatty liver disease is a strong risk factor for insulin resistance and type 2 diabetes [7], but lipid accumulation in liver can be experimentally dissociated from insulin resistance under certain conditions [8], suggesting that other mechanisms are also involved. Liver resident macrophages, called Kupffer cells (KCs), are thought to be the major source of hepatic inflammation [9] and appear to be involved in the regulation of multiple aspects of liver biology [10]. We have previously established a pro-inflammatory function of CB_1_R in macrophages [11] and CB_1_R signaling is strongly involved in the development of fatty liver [12] and insulin resistance [13], [14]. Here, using a method to silence gene expression selectively in Kupffer cells *in vivo* [11], [15], [16], [17], [18], [19], we demonstrate that knock-down of CB_1_R in Kupffer cells leads to improved global insulin sensitivity by reducing inflammation, ROS production and promoting mitochondria uncoupling through an increase in UCP2 activity.

## 2 Material & methods

### 2.1 Animals and diet

The experimental protocol was approved by the Institutional Animal Care and Use Committee of the National Institute on Alcohol Abuse and Alcoholism, National Institutes of Health (NIH). Six-week-old male wild-type C57BL/6 mice were obtained from Taconic Inc. (Taconic, Germantown, NY). *Cnr1*^*−/−*^ mice on a C57Bl/6J background were generated by heterozygote breeding. Mice were maintained under a 12:12-h light–dark cycle and fed *ad libitum* either a high fat diet (HFD, D12492; 60% of calories from fat, 20% from protein, and 20% from carbohydrates; Research Diets, New Brunswick, NJ) or standard mouse chow (NIH-31 rodent diet) for 15 weeks prior to treatment. Food intake was assessed as the cumulative amount eaten over 7 days.

### 2.2 GeRPS administration by intravenous injection *in vivo*

The β1,3-d-glucan-encapsulated siRNA particles (GeRPs) were prepared as previously described [16], [17]. Briefly, diet-induced obese mice were treated with 6 doses of FITC-GeRPs by intravenous injections every 3rd day for 15 days. Each dose of FITC-glucan shells contained 0.33 mg of fluorescently labeled GeRPs loaded with 1 nmol (13.3ug) control (GCAUCAAGUCGACUGUUAA) or *Cnr1* (GCAUCAAGAGCACUGUUAA) siRNA and 16.6 nmol Endoporter.

### 2.3 Glucose homeostasis

Glucose tolerance and insulin sensitivity tests (GTTs and ISTs) were performed 24 h after the last GeRPs injection as described in [20]. A dose of 1.5 g/kg glucose or 0.75 U/kg of insulin was injected intraperitoneally, and blood glucose levels were measured as previously described [20].

### 2.4 Isolation of Kupffer cells and hepatocytes

Liver cells were isolated as described in [17]. Briefly, after anesthesia, the liver was first perfused with calcium-free Hanks balanced salt solution (HBSS, Gibco #14185-052, Gaithersburg, MD) then followed by collagenase digestion (0.6 mg/mL collagenase from *Clostridium histolyticum* [Sigma #C6885, St. Louis, MO] in HBBS containing 1 mM CaCl_2_). After digestion, liver cells were released by dissociation from the lobes and underwent several steps of filtration through a 100 μm cell strainer using ice-cold HBSS-CaCl_2_. Cell suspension was then centrifuged at a speed of 50 g for 3 min at 4 °C. The supernatant from the first centrifugation of hepatocytes was loaded on a Percoll gradient (25% and 50%) and centrifuged for 30 min at 2300 rpm and 4 °C. The interphase ring with Kupffer cells was collected and washed 2 times with PBS. The hepatocyte pellet obtained after the first centrifugation was washed 3 times in the same conditions in order to obtain the enriched hepatocyte fraction. Cells were cultured overnight in RPMI-1640 medium (ThermoFisher Scientific, 11875093, Waltham, MA) supplemented with 10% FBS (ThermoFischer Scientific, 10082147, Waltham, MA), 100 nM dexamethasone (Sigma #D-4902, St. Louis, MO), 100 nM insulin (Gibco #12585-014, Gaithersburg, MD), and 1% Penicillin/Streptomycin (ThermoFischer Scientific, 15140122, Waltham, MA) at 37 °C and 5% CO2. The following day, primary cells were used for subsequent analyses.

### 2.5 Preparation of conditioned medium

Hepatocyte conditioned medium (CM) was prepared by incubating primary hepatocytes isolated from lean or diet-induced obese (DIO) mice for 48 h in the condition described above. KCs CM was obtained by incubating primary KCs isolated from WT or global *Cnr1*^*−/−*^ mice for 48 h in the same medium described above in the presence of 20 μg/mL of LPS.

### 2.6 Serum parameters

Serum insulin was measured using the STELLUX™ Chemi Rodent Insulin ELISA (ALPCO, Salem, NH), c-peptide and adiponectin were quantified using ELISA kits according to manufacturer's instruction (ALPCO, Salem, NH). Circulating ALT, AST, triglycerides and total cholesterol were quantified by colorimetric kits from BioAssay Systems (Hayward, CA).

### 2.7 Liver parameters

Intrahepatic triglyceride content was determined as previously described [11] whereas glycogen content was determined based on the enzymatic reaction described in [21]. Tissue and cells extraction for endocannabinoids measurement by liquid chromatography–tandem mass spectrometry (LC-MS/MS) was performed as previously described [11].

### 2.8 Immunohistochemistry

KCs were identified in liver and adipose tissue sections using antibodies against F4/80 (AbD Serotec, Raleigh, NC) or Iba-1 (Wako, 019-1974) and analyzed using a Zeiss LSM700 confocal microscope. Immunopositivity was quantified using Image J software.

### 2.9 Reactive Oxygen Species (ROS) detection

ROS production in isolated KCs was determined with the DCFDA – Cellular Reactive Oxygen Species Detection Assay Kit (Abcam, Ab113851, Cambridge, UK) according to manufacturer's instructions.

### 2.10 Assays for NF-κB p65 phosphorylation

Phosphorylation of NF-κB p65 in KCs was assessed by a phospho-RelA/NF-κB p65 ELISA kit (R&D Systems, KVB7226, Minneapolis, MN) according to the manufacturer's instructions.

### 2.11 Immunoblotting

Cell lysis was performed in 1x RIPA buffer (Thermo-Scientific) supplemented with phosphatase and protease inhibitor tablets (Roche), 2 mM Na_3_VO_4_, and 2 mM NaF. Total protein concentration was determined with BCA assay (Thermo-Scientific) and adjusted to the same concentration with additional lysis buffer. Proteins were separated on SDS-PAGE (4–12% Bis-Tris or Tris-Glycin gradient gel, Bio-Rad, Hercules, CA) and transferred onto nitrocellulose membranes. Membranes were blocked in 3% non-fat milk in Tris-buffered saline (TBS) with 0.1% Tween-20 (TBST) and incubated overnight at 4 °C in 5% BSA TBST with primary antibody against phospho-p65 (RelA), p65, phospho-AKT^Ser473^, AKT (Cell Signaling #9271, #9272, 1/1000, Danvers, MA, USA), UCP2 (Cell Signaling #89326, Danvers, MA), FABP4 (Abcam, Ab66682), CB_1_R (rabbit polyclonal antibody, Immunogenes, Hungary), or HRP-conjugated mouse monoclonal antibody anti β-actin (Abcam ab49900, 1/20, 000) as a loading control. Appropriate secondary antibody was diluted in 5% non-fat milk TBST and incubated for 1 h at room temperature. Luminescent signal was generated with Super Signal West Pico chemiluminescent substrate (Thermo-Scientific) and detected with a membrane imager (G:BOX, Syngene). Quantification was performed using the analysis options of NIH Image J software.

### 2.12 Real-time PCR

Total RNA extraction from liver, hepatocytes and Kupffer cells was reverse transcribed, and real-time PCR were performed as previously described [11]. QuantiTect Primer Assays (Qiagen, Germantown, MD) were used to detect gene expression. Expression of a gene of interest is reported as a relative value comparing it to the geometric average of 18S, L19, L38, and TATA box binding protein expression.

### 2.13 Statistics

Values are expressed as means ± SEM. Data were analyzed by Student's t-test (GraphPad Prism v6 for Windows). Significance was set at P < 0.05.

## 3 Results

### 3.1 *In vivo Cnr1* knock-down in KCs improves glucose tolerance and insulin sensitivity without affecting body weight or liver fat content in obese mice

**Figure 1:**
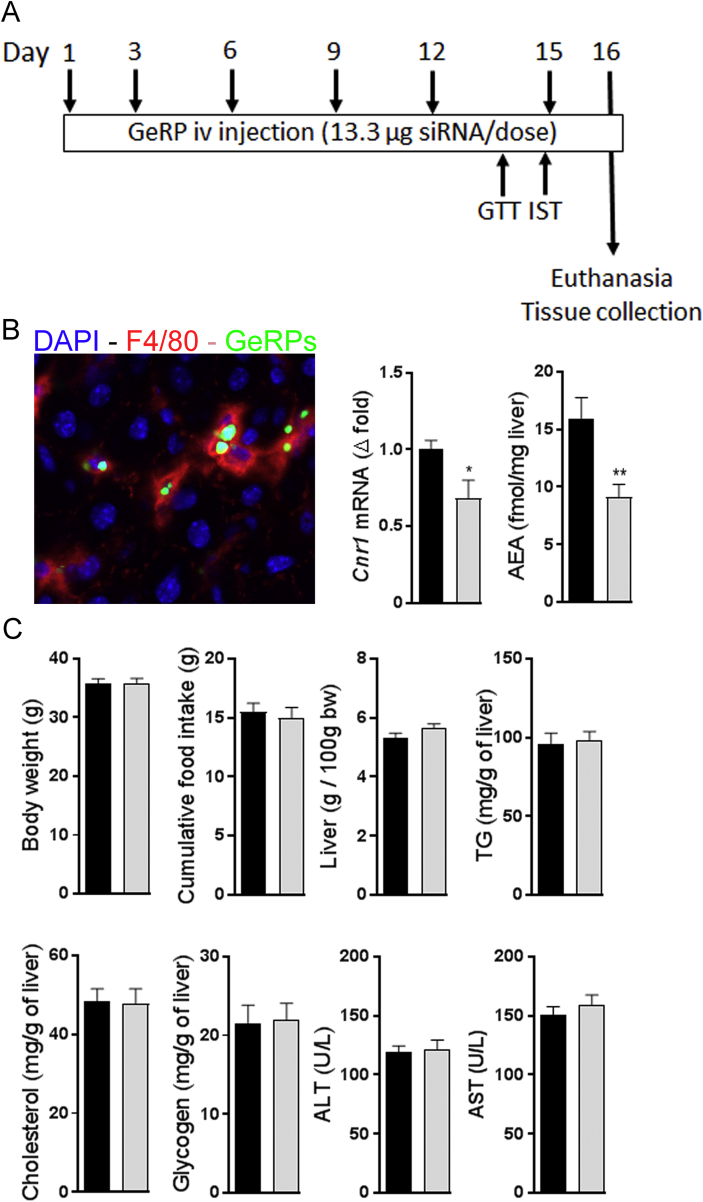
**GeRP-mediated selective knock-down of *Cnr1* in Kupffer cells in obese mice does not influence hepatic steatosis**. **A** Protocol for 15-day GeRP treatment in obese mice. **B** KCs staining (red) in liver section from an obese mouse 24 h after intravenous injection with FITC-labeled GeRPs (green) (magnification ×200). *Cnr1* gene expression and AEA content in liver from DIO mice treated with control (black columns, n = 10) or *Cnr1*-GeRPs (light grey columns, n = 10). **C** Body weight, cumulative food intake, liver weight, and liver TG, cholesterol, and glycogen content in obese mice treated with control or *Cnr1*-GeRPs. Columns and bars represent means ± SEM. Significant differences from values in control-GeRPs treated obese mice *P < 0.05, **P < 0.01, ***P < 0.001.

**Figure 2:**
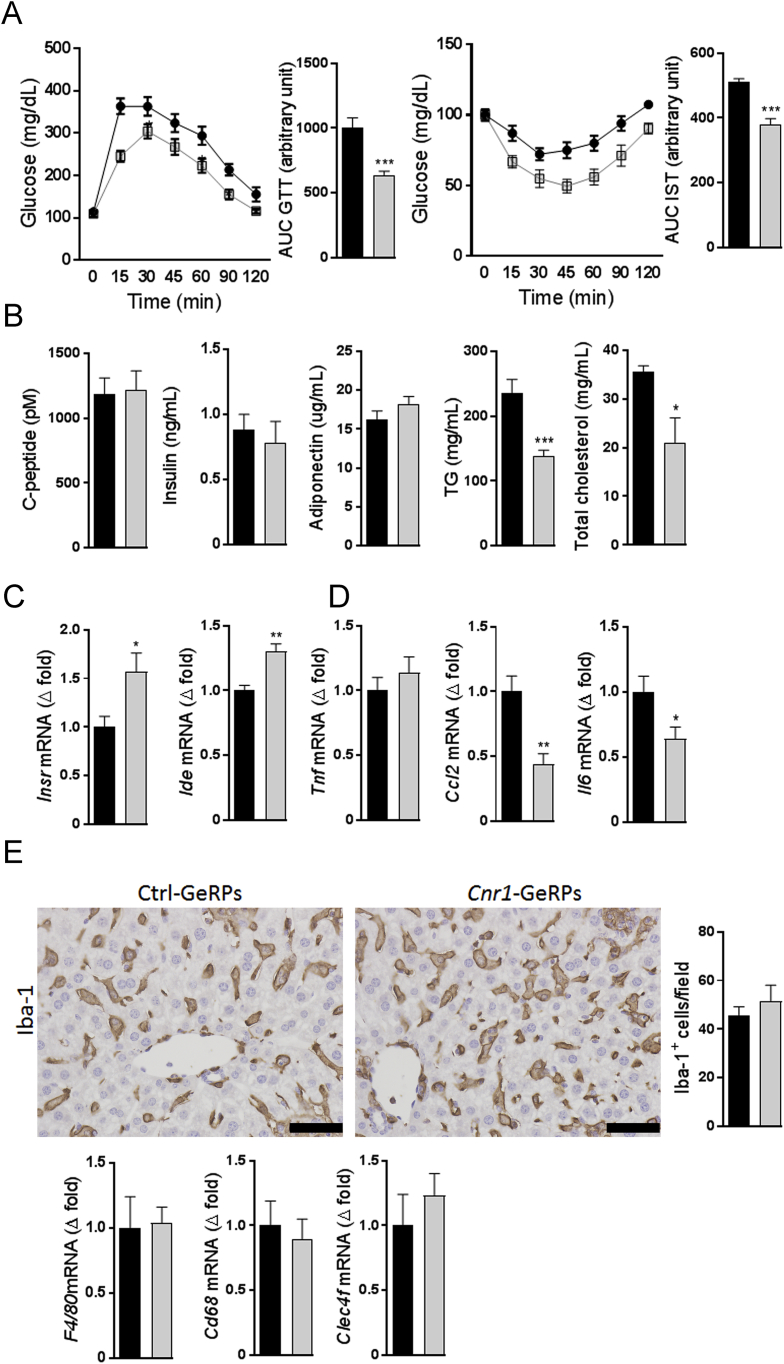
***Cnr1* knock-down in Kupffer cells of DIO mice improves glucose tolerance, insulin sensitivity and reduces hepatic inflammation. A** Glucose tolerance (GTT) and insulin sensitivity test (IST) from DIO mice treated with control (black columns, n = 10) or *Cnr1*-GeRPs (light grey columns, n = 10). Points and bars are means ± SEM from 2 independent experiments. Areas under the curve (AUC) from each experiment were used for statistical analyses. **B** Plasma concentrations of c-peptide, insulin, adiponectin triglycerides, and total cholesterol. **C** Whole liver gene expression of insulin receptor (*Insr*) and insulin-degrading enzyme (*Ide*). **D** Whole liver gene expression of the pro-inflammatory cytokines *Tnf*, *Ccl2* and *Il-6*. **E** Hepatic macrophage histology as assessed by Iba-1 immuno-staining (scale bars: 50 μm) and whole liver gene expression of macrophage markers *F4/80*, *Cd68*, and *Clec4f*. Columns and bars represent means ± SEM. Significant differences from values in control-GeRPs treated obese mice *P < 0.05, **P < 0.01, ***P < 0.001.

Diet-induced obese (DIO) mice were treated with fluorescently labeled GeRPs via intravenous injections every 3rd day for 15 days (Figure 1A), resulting in their selective uptake by KCs (Figure 1B). In agreement with previous observations [17], Using GeRPs containing a siRNA against *Cnr1* resulted in a significant, ∼33% decrease in global liver *Cnr1* expression and a ∼50% decrease in hepatic anandamide levels (Figure 1B). This treatment had no impact on body weight, food intake, liver mass, or hepatic triglycerides, cholesterol, and glycogen content (Figure 1C). Similarly, no differences were observed in terms of circulating ALT and AST levels between groups (Figure 1C). However, mice receiving *Cnr1* siRNA displayed improved glucose tolerance and insulin sensitivity (Figure 2A), whereas no differences between the groups were noted regarding circulating insulin, c-peptide and adiponectin (Figure 2B). Interestingly, siRNA-mediated *Cnr1* knock-down in KCs led to a significant reduction in circulating triglycerides and total cholesterol levels (Figure 2B) and also increased hepatic insulin receptor (*Insr*) and insulin degrading enzyme (*Ide*) gene expression (Figure 2C). Moreover, this treatment was associated with a lower expression of *Ccl2* and *Il6* but not *Tnf*, suggesting a decrease in liver inflammation (Figure 2D). Interestingly, the number of Iba-1 positive hepatic macrophages was similar in the 2 groups. Similarly, the gene expression of three KCs markers, *F4/80*, *Cd68* and C-type lectin domain family 4 member F (*Clec4f*), remained unaffected (Figure 2E), suggesting that the reduced inflammation was not due to a decrease in hepatic macrophage content. Also, intravenous injection of GeRPs did not influence the number of macrophages present in adipose tissue (Supplementary Figure 1A) and did not influence *Cnr1* expression is this tissue (Supplementary Figure 1B).

### 3.2 *In vivo Cnr1* knock-down in KCs reduces endocannabinoid tone selectively in KCs

**Figure 3:**
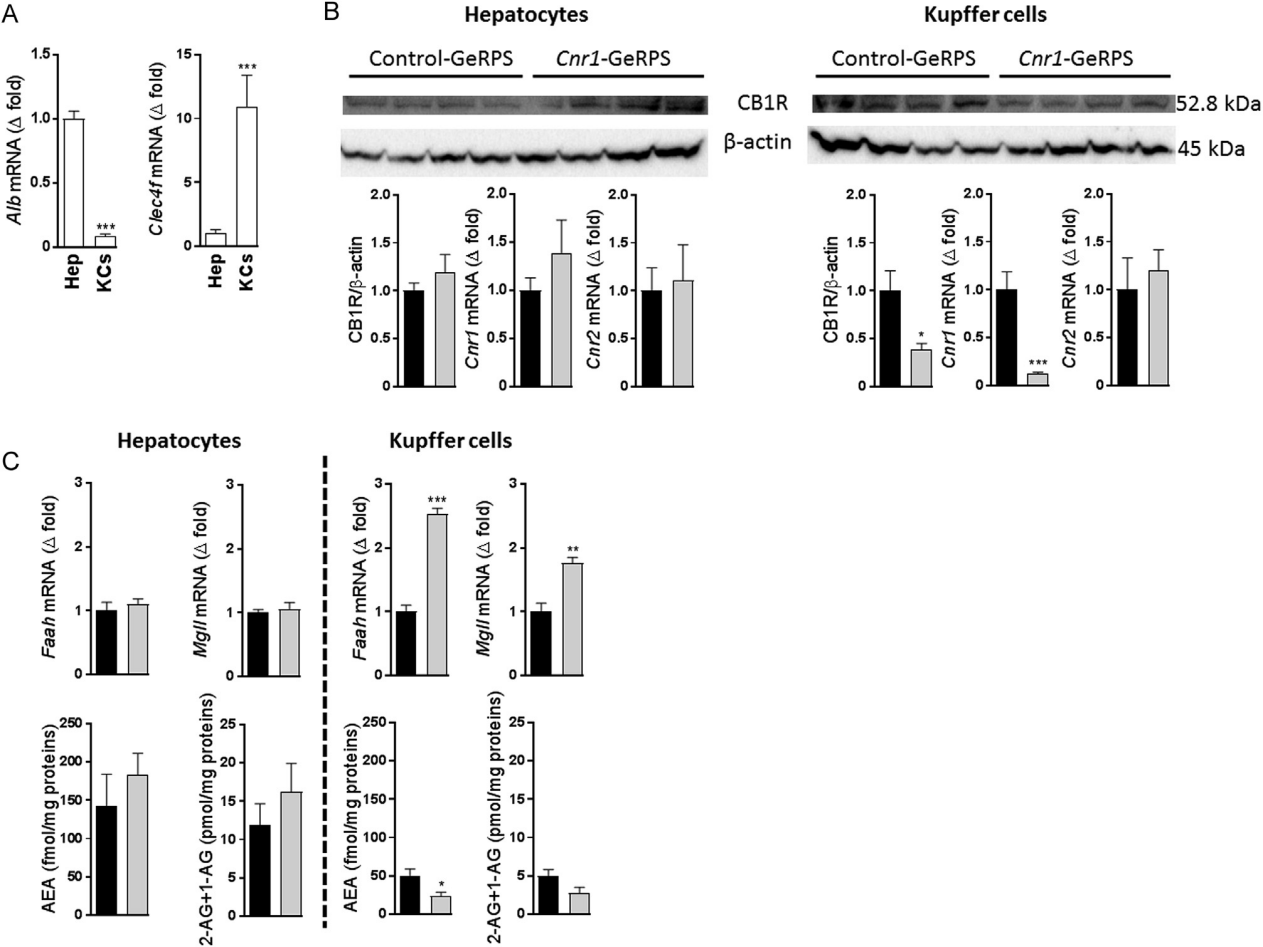
***In vivo Cnr1* knock-down in Kupffer cells reduces the endocannabinoid tone in these cells. A** Relative expression of *Alb* and *Clec4f* in hepatocyte and Kupffer cell fractions, with levels in hepatocytes defined as 1.0. **B** CB_1_R protein quantification by western blot and *Cnr1*/*Cnr2* gene expression in hepatocytes or Kupffer cells isolated from DIO mice treated with control (black columns) or *Cnr1*-GeRPs (grey columns). **C** Gene expression for *Faah* and *Mgll* and endocannabinoid (AEA and 2-AG) quantification in hepatocyte and Kupffer cell fractions from DIO mice treated either with control (black columns) or *Cnr1*-GeRPs (grey columns). Columns and bars represent means ± SEM. Significant differences from values in DIO mice treated with control-GeRPs, *P < 0.05, **P < 0.01, ***P < 0.001.

Hepatocyte and KC fractions were isolated from the liver of mice sacrificed 24 h following the administration of GeRPs. The purity of the fractions was analyzed by the expression of the hepatocyte marker albumin (*Alb*) and the KC marker *Clec4f* [17], which showed an enrichment of over 80% for the respective fractions **(**Figure 3A). As expected, *Cnr1* expression remained unchanged by GeRP treatment in hepatocytes as these cells did not contain GeRPs, whereas it was reduced by 80% in KCs, which was paralleled by a nearly 60% reduction in CB_1_R protein levels as compared to KCs isolated from mice treated with control GeRPs (Figure 3B). The much greater reduction of *Cnr1* in KCs compared to whole liver (see Figure 1B) is likely due to the expression of *Cnr1* in other liver cell types such as hepatocytes [22], stellate cells [23], cholangiocytes [24], and liver vascular endothelial cells [25], which remained unaffected due to lack of uptake of GeRPs by these cells [15], [17]. Additionally, *Cnr1* knock-down was associated with an increase in *Faah* and *Mgll* mRNA, which encode endocannabinoid degrading enzymes, along with a reduced anandamide and 2-arachidonoyl-glycerol content in KCs but not in hepatocytes (Figure 3C).

### 3.3 *In vivo Cnr1* knock-down in KCs restores insulin signaling and induces a shift from pro-inflammatory M1 to anti-inflammatory M2 profile

**Figure 4:**
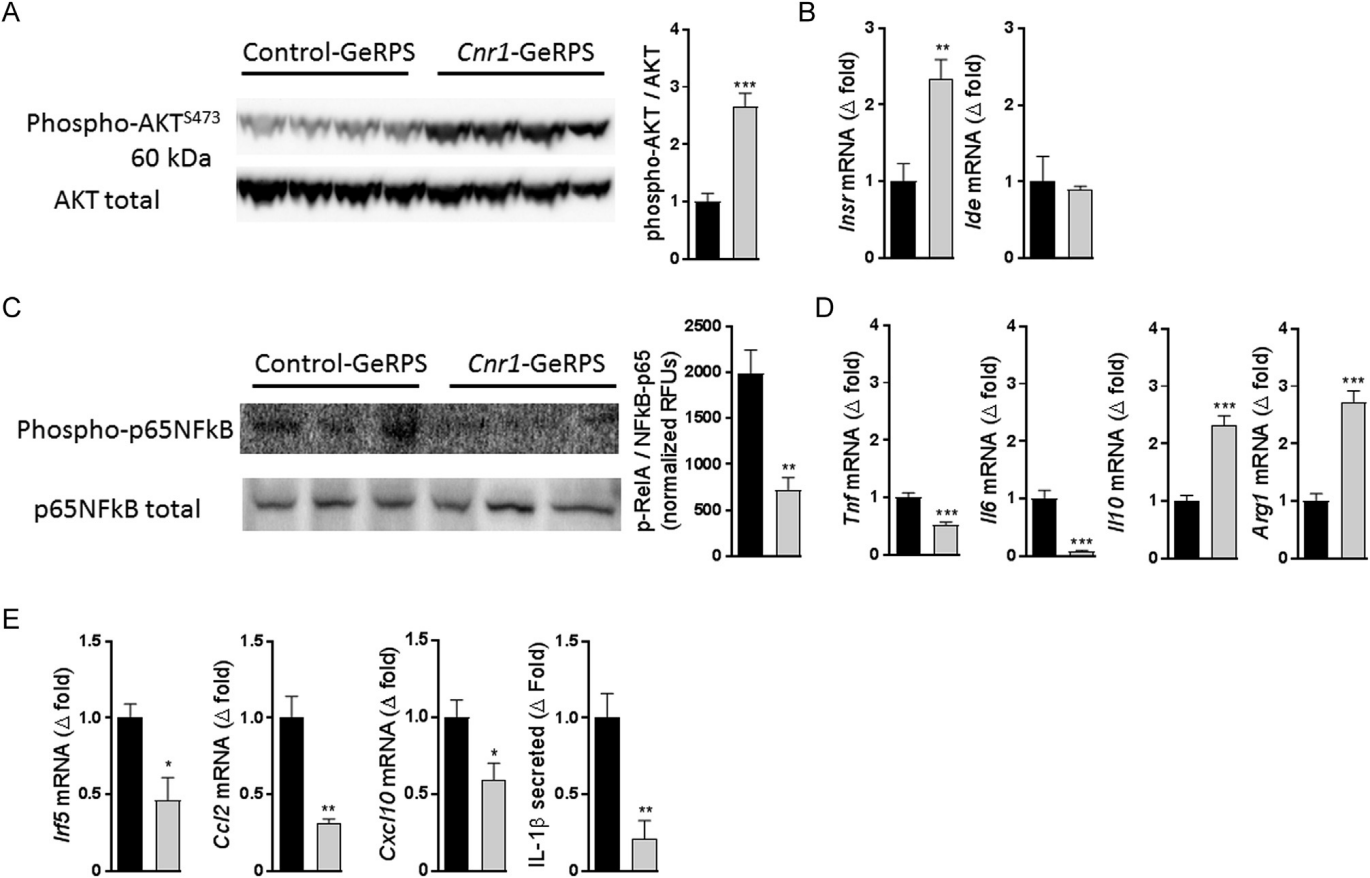
***In vivo Cnr1* knock-down in KCs restores insulin signaling and promotes an anti-inflammatory profile. A** Representative Akt phosphorylation by western blot after insulin stimulation and its quantification. **B** Gene expression of insulin receptor (*Insr*) and insulin degrading enzyme (*Ide*) in isolated Kupffer cells. **C** Representative NF-κB phosphorylation analysis by western blot and by cell-based ELISA kit. **D** Gene expression for TNFα (*Tnf*), IL-6 (*Il6*), IL-10 (*Il10*), and arginase 1 (*Arg1*) in isolated Kupffer cells. **E** Gene expression for interferon regulatory factor 5 (*Irf5*), CCL2 (*Ccl2*), CXCL10 (*Cxcl10*), and secretion of IL-1β from isolated Kupffer cells from obese mice treated either with control (black columns) or *Cnr1*-GeRPs (grey columns). Columns and bars represent means ± SEM. Significant differences from values in control-GeRPs treated DIO mice *P < 0.05, **P < 0.01, ***P < 0.001.

Similar to the improved insulin sensitivity following the treatment of DIO mice with peripheral CB_1_R antagonists [20], [26], insulin-stimulated Akt^S473^ phosphorylation was increased in KCs of mice treated with *Cnr1*-GeRPs compared to control GeRPs (Figure 4A). KCs with *Cnr1* knock-down displayed an increase in insulin receptor (*Insr*) but not in insulin degrading enzyme (*Ide*) mRNA expression (Figure 4B). *Cnr1* knock-down was associated with an inhibition of NF-kB activity, as reflected by the reduced p65 phosphorylation observed by western blotting and by cell-based ELISA (Figure 4C). In parallel, a shift from a pro-to anti-inflammatory phenotype was indicated by lower *Tnf* and *IL6* as well as higher *Il10* and Arginase (*Arg1*) expression (Figure 4D). Additionally, KCs with reduced *Cnr1* expression also had a marked reduction in *Irf5*, *Ccl2*, and *Cxcl10* expression along with reduced Il-β protein secretion (Figure 4E).

### 3.4 *In vivo Cnr1* knock-down reduces oxidative stress markers and increases mitochondria uncoupling in KCs

**Figure 5:**
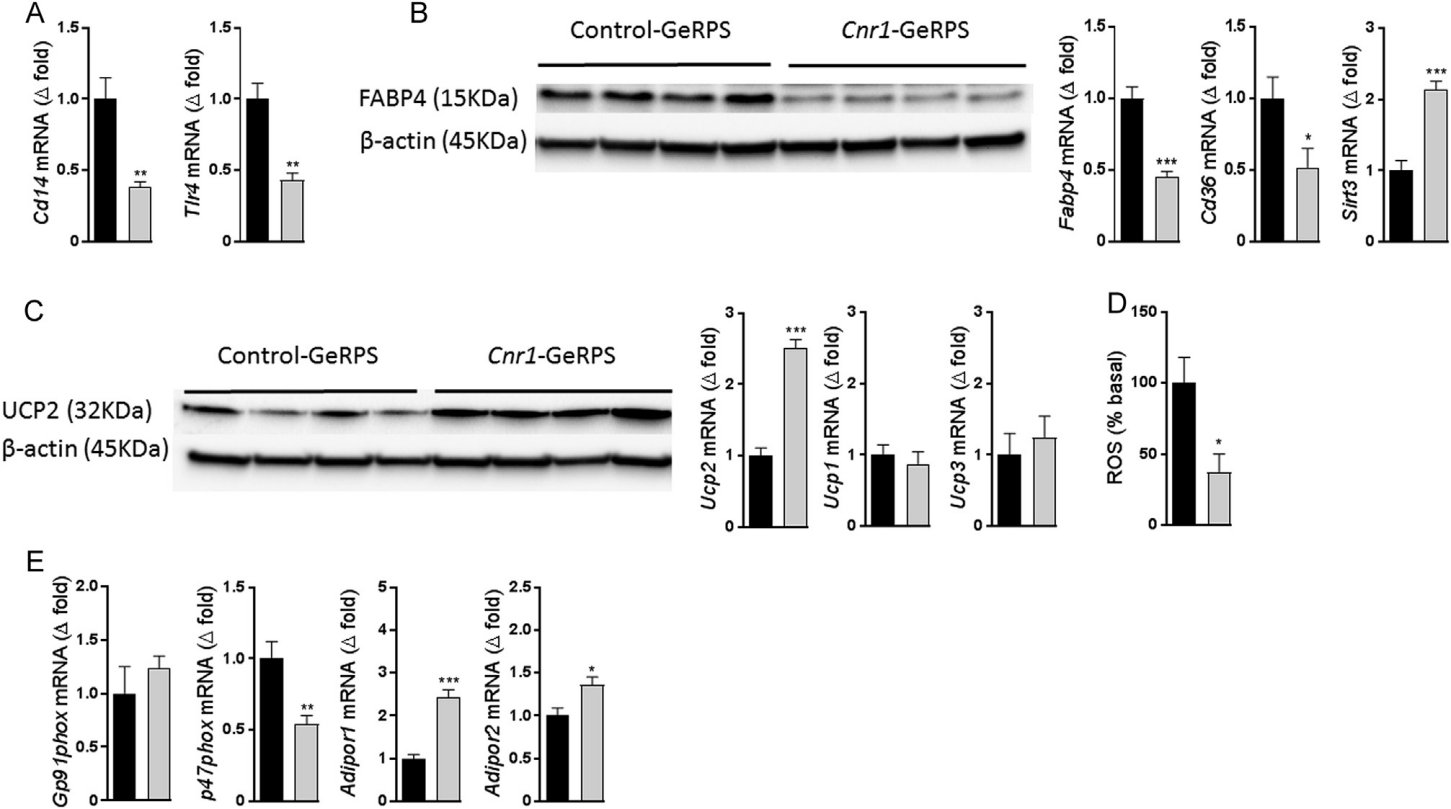
***In vivo Cnr1* knock-down in KCs reduces oxidative stress. A** Gene expression for CD14 and TLR4 in Kupffer cells. **B** Representative western blot showing FABP4 protein levels in Kupffer cells isolated from obese mice treated with either control or *Cnr1*-GeRPs and gene expression for *Fabp4* and Sirtuin-3 (*Sirt3*). **C** UCP-2 protein and gene expression along with *Ucp1* and *Ucp3* gene expression in Kupffer cells. **D** Reactive Oxygen Species (ROS) produced by Kupffer cells isolated from obese mice treated with either control or *Cnr1*-GeRPs. **E** Gene expression for *Gp91phox*, *p47phox*, *AdipoR1*, and *Adipor2* in isolated Kupffer cells from obese mice treated either with control (black columns) or *Cnr1*-GeRPs (grey columns). Columns and bars represent means ± SEM. Significant differences from values in control-GeRPs treated obese mice *P < 0.05, **P < 0.01, ***P < 0.001.

*Cnr1* knock-down in KCs resulted in reduced expression of *Cd14* and *Tlr4*, genes encoding two receptors involved in NF-κB activation (Figure 5A), along with downregulation of the fatty acid binding protein-4 both at the mRNA and protein levels as well as a downregulation of *Cd68* and upregulation in sirtuin 3 (*Sirt3*) (Figure 5B). In parallel, uncoupling protein 2 (UCP2) mRNA and protein were elevated in KCs with *Cnr1* knock-down, whereas *Ucp1* and *Ucp3* expression did not change (Figure 5C). Together, these data suggest a link between CB_1_R signaling and mitochondria uncoupling in KCs. Knock-down of *Cnr1* also led to a significant decrease in reactive oxygen species (ROS) activity, as quantified using 2′,7′–dichlorofluorescin diacetate (DCFDA) as a probe (Figure 5D). Moreover, *Cnr1* knock-down reduced the expression of *Gp91phox* and *p47phox*, 2 subunits of the multi-protein complex NADPH oxidase 2, and increased *Adipor1* and *Adipor2* mRNA expression, suggesting a decrease in oxidative stress (Figure 5E).

### 3.5 Hepatocyte-derived endocannabinoids regulate cytokine secretion by KCs

**Figure 6:**
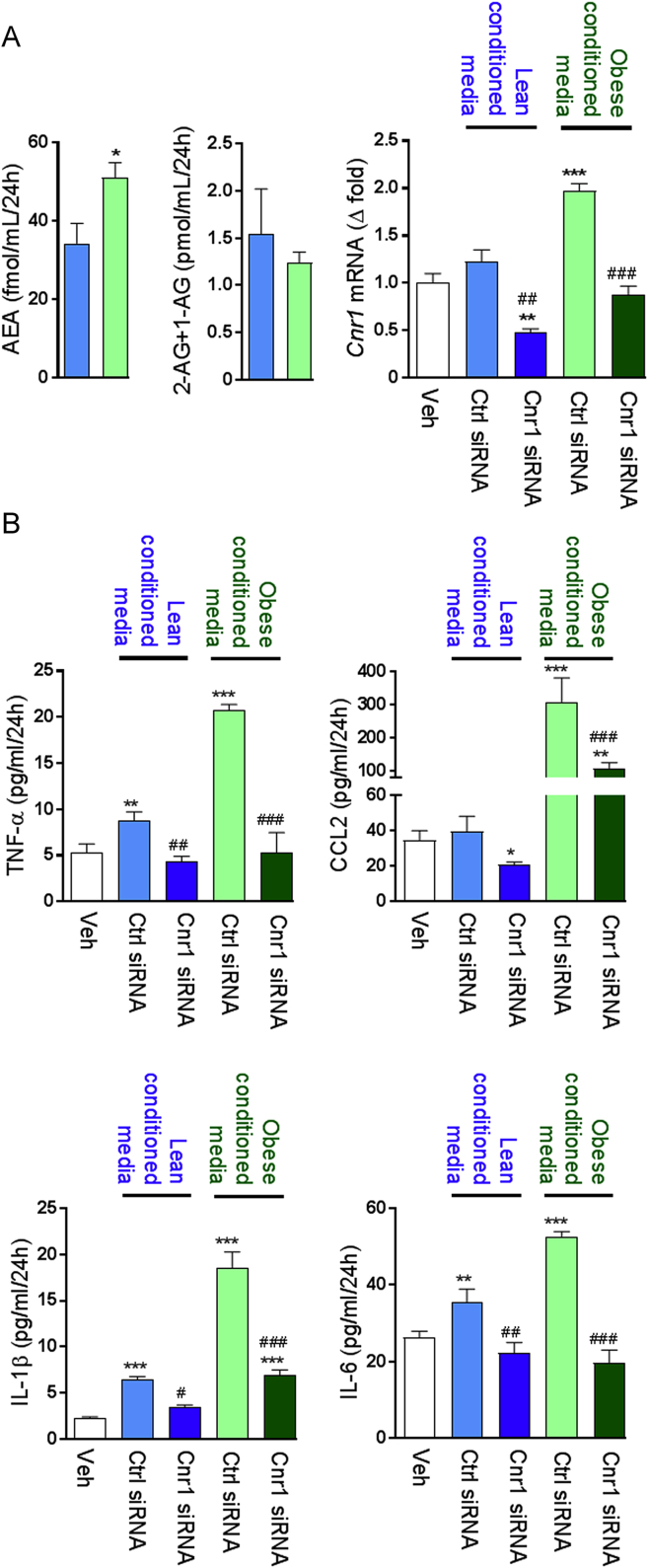
**KCs secretary response is influenced by changes in endocannabinoid tone. A** Endocannabinoid content in conditioned medium from hepatocytes isolated from lean (blue columns) or obese mice (green columns) and *Cnr1* expression in Kupffer cells incubated for 24 h in these conditioned media in presence of either control or *Cnr1* siRNA. **B** Effect of conditioned medium on Kupffer cells secretion of TNF-α, CCL2, IL-1β, and IL-6 after 24 h. Columns and bars represent means ± SEM from 3 individual experiments with n = 4 per condition. Significant differences from values in Veh-treated Kupffer cells (white columns) *P < 0.05, **P < 0.01, ***P < 0.001 or control siRNA treated cells (blue or green columns) #P < 0.05, ##P < 0.01, ###P < 0.001.

In order to investigate the stimuli involved in the inflammatory response of KCs, we incubated KCs isolated from lean mice in presence of conditioned medium (CM) from hepatocytes isolated from lean or obese mice. The CM from obese hepatocytes contained more anandamide (AEA) but not 2-arachidonoyl glycerol (2-AG) than CM from lean hepatocytes, and it triggered an increase in *Cnr1* in lean KCs which could be prevented by *Cnr1*-siRNA pre-treatment (Figure 6A). KCs incubated with the CM from obese hepatocytes secreted increased amounts of TNFα, CCL2, IL-1β, and IL-6 compared to KCs incubated with CM from lean hepatocytes (Figure 6B), and these changes were CB_1_R-dependent as they were abrogated by *Cnr1* knock-down (Figure 6B).

### 3.6 Changes in inflammatory tone directly influence hepatocytes response to insulin

**Figure 7:**
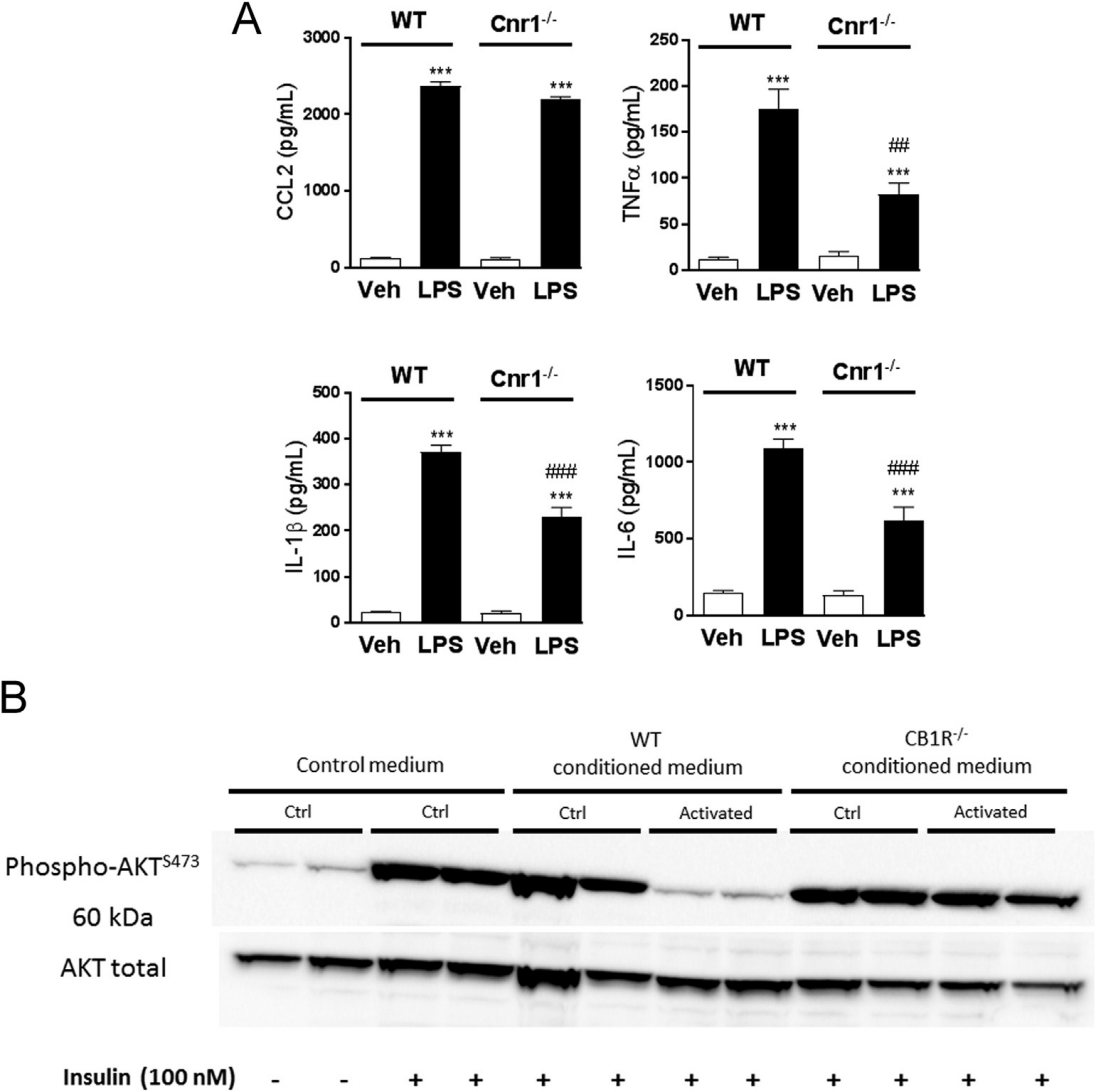
**Changes in inflammatory tone directly influence hepatocyte response to insulin. A** Cytokine and chemokine production by Kupffer cells isolated from wildtype (WT, empty columns) or CB_1_R^−/–^ mice (*Cnr1*^−/−^, black columns) after 48 h of treatment with saline or LPS (50 ng/mL). Columns and bars represent means ± SEM from 3 individual experiments with n = 3 replicates per condition. **B** Representative western blot showing Akt phosphorylation in response to insulin challenge (100 nM, 30 min) in hepatocytes incubated for 24 h in conditioned medium obtained from LPS-treated Kupffer cells as described in **A**. Columns and bars represent means ± SEM. Significant differences from values in vehicle treated cells *P < 0.05, **P < 0.01, ***P < 0.001 or LPS treated cells #P < 0.05, ##P < 0.01, ###P < 0.001.

In view of the EC-mediated increase in pro-inflammatory cytokine secretion by KCs, we investigated if this could impair the insulin response of hepatocytes. KCs isolated from WT and CB_1_R^−/–^ mice were incubated with vehicle or LPS (50 ng/mL) for 48 h. LPS triggered an increase in TNFα, IL-1β, and IL6 but not in CCL2 secretion, which was much stronger in WT compared to CB_1_R-deficient KCs (Figure 7A). We then incubated primary hepatocytes from lean mice with regular medium or KC conditioned medium and tested their response to insulin. Insulin induced a robust increase in AKT phosphorylation on Ser^473^ in hepatocytes maintained in regular medium or in CM from CB_1_R-deficient KCs, but its effect was abrogated in hepatocytes incubated with CM from WT KCs (Figure 7B), suggesting that a CB_1_R-mediated cytokine secretion by KCs inhibits hepatocyte insulin signaling.

## 4 Discussion

In the present study, we demonstrated that selective silencing *Cnr1* in KCs improves hepatic insulin sensitivity in DIO mice, independent of ectopic fat deposition. We used the GeRP technology to deliver siRNA to KCs without affecting other hepatic cells [15], [16], [17], [18], [19] by taking advantage of the micrometer-size and Dectin 1 receptor-mediated recognition of the glucan shells [16]. Further specificity for hepatic macrophages was ensured by using the intravenous route for the administration of GeRPs, which limits their distribution to the liver without affecting macrophages in other tissues including adipose tissue [17]. As resident macrophages, KCs are the major source of pro-inflammatory cytokines in the liver [27], [28], and steatotic hepatocytes influence the M1/M2 balance of KCs by promoting the apoptosis of alternatively-activated M2 KCs, thus shifting their balance toward the pro-inflammatory M1 phenotype [29]. CD14 has been reported as a potential marker for necrotic liver inflammation, and its expression correlates with the phagocytic function of KCs [30]. Moreover, binding of LPS to CD14 on the KC membrane activates IKK kinases, thus relieving the IκB-mediated inhibition of NF-κB, leading to an increase in TNF-α secretion [31]. This, in turn, triggers monocyte infiltration through the expression of C-X-C motif chemokine 10 (CXCL10) and Chemokine ligand 2 (CCL2) [32]. In addition, increases in the levels of such pro-inflammatory cytokines in patients with non-alcoholic steatohepatitis (NASH) are related to the level of CD14^+^ KCs [27], [33], [34]. In view of the above, the decreased expression of *Cd14*, *Tnf*, *Cxcl10*, and *Ccl2* in KCs following *Cnr1* knock-down reflects a decrease in their pro-inflammatory polarization.

KCs are also activated by the binding of LPS or FFA to TLRs resulting in the release of cytokines and chemokines via NF-κB signaling. The activation of the NF-κB signaling pathway is also directly induced by oxidative or endoplasmic reticulum (ER) stress. Several pro-inflammatory cytokines, such as TNF-α, IL-1β, IL-6, C-C chemokine receptors 2 (CCR-2), macrophage inflammatory protein 1 (MIP-1), COX-2, CCL2, and intercellular adhesion molecule/vascular cell adhesion molecule (ICAM/VCAM) are produced by the activated NF-κB pathway [35], [36], and systemic inhibition of NF-κB reduces hepatic inflammation and improves insulin resistance [37]. Here, we found a drastic inhibition of NFκB activation after *Cnr1*-knockdown in KCs, partially explaining the associated decrease in the expression of pro-inflammatory markers such as *Tnf*, *Ccl2*, *Cxcl10*, *Il6*, and IL-1β. The degree of NF-κB inhibition and the associated reduction in cytokine release and insulin sensitization following *Cnr1* knock-down were similar to those reported following a KC-specific knock-down of NF-κB [17]. This suggests that CB_1_Ris a major upstream signal involved in NF-κB activation in KCs.

The transcription factor interferon regulatory factor 5 (*Irf5*) plays an important role in polarizing macrophages towards an inflammatory phenotype and promoting insulin resistance and hepatic fibrosis [38], [39], [40], [41]. Thus, the 50% decrease in *Irf5* mRNA expression induced by *Cnr1* knockdown in KCs likely contributes to their M1→M2 shift and to the improved *in vivo* insulin sensitivity. This is in agreement with our previous findings showing that IRF5 is a downstream target of CB_1_R in macrophages, in which it drives the CB_1_R-mediated increase in TNF-α secretion, and its *in vivo* knock-down in macrophages prevents the loss of pancreatic islet β-cells and the development of type 2 diabetes [42]. Our observations linking *Irf5* expression and CB_1_R signaling are also in agreement with findings that *IRF5* expression in obese subjects is negatively correlated with insulin sensitivity [38], [39], [40].

Adiponectin is known to stimulate the production of IL-10 and IL-1R antagonist, decrease phagocytic activity, and suppress pro-inflammatory cytokine production in macrophages by inhibiting NF-κB [43], [44], [45]. Adiponectin can also promote macrophage polarization toward an anti-inflammatory M2 phenotype [46], [47] similarly to *Cnr1* knock-down. In addition, this effect can be either direct [46] or indirect through IL-4-mediated M2 polarization [48]. Accordingly, we found that *Cnr1* knockdown in KCs led to increased expression of both *Adipor1* and *Adipor2,* which was associated with increased *Il10* and *Arg1* expression. *AdipoR1* is abundantly expressed in macrophages [44], [49], [50], whereas *AdipoR2* is predominantly expressed in the liver [51]. AdipoR1 signals via activating AMP-activated kinase (AMPK) while AdipoR2 activates PPARα, both contributing to increased insulin sensitivity [52]. Simultaneous disruption of both AdipoR1 and AdipoR2 abolished adiponectin binding and actions, resulting in increased liver triglyceride content, inflammation and oxidative stress in adipose tissue, and consequent insulin resistance and glucose intolerance [53]. A similar mechanism could be at play in KCs following *Cnr1* knock-down, as indicated by the increase in both *Adipor1* and *Adipor2* expression. Indeed, macrophage-specific *AdipoR1* transgenic mice (AdR1-TG) exhibit enhanced whole-body glucose tolerance and insulin sensitivity with reduced pro-inflammatory cytokines, MCP-1 and TNF-α, both in the serum and in the insulin target metabolic tissues [54].

Inflammation is often coupled to increased ROS production and a pivotal source of ROS in inflammatory cells is the NADPH oxidase or NOX [55]. Rapid release of ROS in response to LPS and other microbial stimuli in KCs and other macrophages occurs mainly through the isoform NOX2 [56]. Upon activation, NOX2 will produce superoxide, a major form of ROS that signals to redox-sensitive targets [57], [58], [59]. UCP2 has been considered in the pathogenesis of NAFLD since its identification [60], [61]. In this condition, KCs and other macrophages have diminished UCP2, which could be attributed to the increased oxidative stress seen in fatty liver [62]. In our study, we observed that *Cnr1* knock-down in KCs led to an increase in *Ucp2* expression without any changes in *Ucp1* or *Ucp3* expression, suggesting that the reduction in ROS production we observed was due, at least partially, to the uncoupling of the mitochondrial respiratory chain, especially since macrophages isolated from Ucp2^−/−^ mice were reported to generate more ROS than those from wild-type mice [63]. The observed reduction in the expression of *Fabp4* in CB_1_R-deficient KCs can also contribute to reduced oxidative stress, as it was recently shown that inhibition or genetic deletion of FABP4/aP2 in macrophages caused an increase in UCP2, resulting in decreased ER stress and a UCP2-dependent reduction in ROS production [64]. This is in agreement with previous findings where *Leishmania donovani* infection in macrophages was associated with a strong upregulation of UCP2 and a strong reduction in ROS generation [65]. Furthermore, in agreement with our findings, it was recently shown that lower FABP4 can induce *Sirt3* expression, which was linked to a decrease in ROS production and an anti-inflammatory phenotype in macrophages [66].

Finally, experiments using conditioned media from LPS-stimulated KCs implicated endocannabinoids in triggering the secretion of pro-inflammatory cytokines by KCs, which then could act on neighboring hepatocytes to inhibit insulin signaling. Previously, we have demonstrated that LPS causes a robust, >10-fold increase in anandamide synthesis in RAW264.7 macrophages [67], which could act as autocrine mediator on CB_1_R located on the same cell. Indeed, the involvement of CB_1_R in cytokine release by KCs is supported by the reduced cytokine release and corresponding loss of biological activity of medium conditioned with CB_1_R-deficient KCs, as illustrated in Figure 7. These observations suggest a cross-talk between KCs and hepatocytes, which could trigger hepatic insulin resistance.

To conclude, the data presented demonstrate that CB_1_R signaling in KCs plays an important role in hepatic insulin resistance independently of ectopic fat in the liver or adipose tissue inflammation. Given the documented role of hepatocyte CB_1_R in insulin resistance [14], our study reinforces the importance CB_1_R expressed by different types of liver cells in glycemic control and further highlights the therapeutic potential of peripheral CB_1_R blockade in the metabolic complications of obesity.

## Author contributions

T.J. and G.K. designed the study, analyzed results and wrote the manuscript; T.J. performed most of the experiments; Z.Z. performed all liver perfusion and helped isolating hepatocytes and Kupffer cells; S.N. and Y.S. prepared, provided, designed, and tested the GeRPs; J.L. and N.J.C. assisted with cell culture, western-blot and PCR analysis; R.C. conducted the endocannabinoids measurements; G.G. assisted with animal care and *in vivo* experiments; M.A. and M.P.C. provided insights and critical review of the manuscript; B.G. provided helpful and critical comments on the manuscript. All authors had access to the manuscript and agreed with the final version.
