# Supplementary material for: Decreasing CB1 receptor signaling in Kupffer cells improves insulin sensitivity in obese mice
Source: Mol Metab. 2017 Sep 1;6(11):1517–28. doi: 10.1016/j.molmet.2017.08.011 (PMC5681272; doi:10.1016/j.molmet.2017.08.011)
Supplement: Supplementary file 1 [file mmc1.docx]

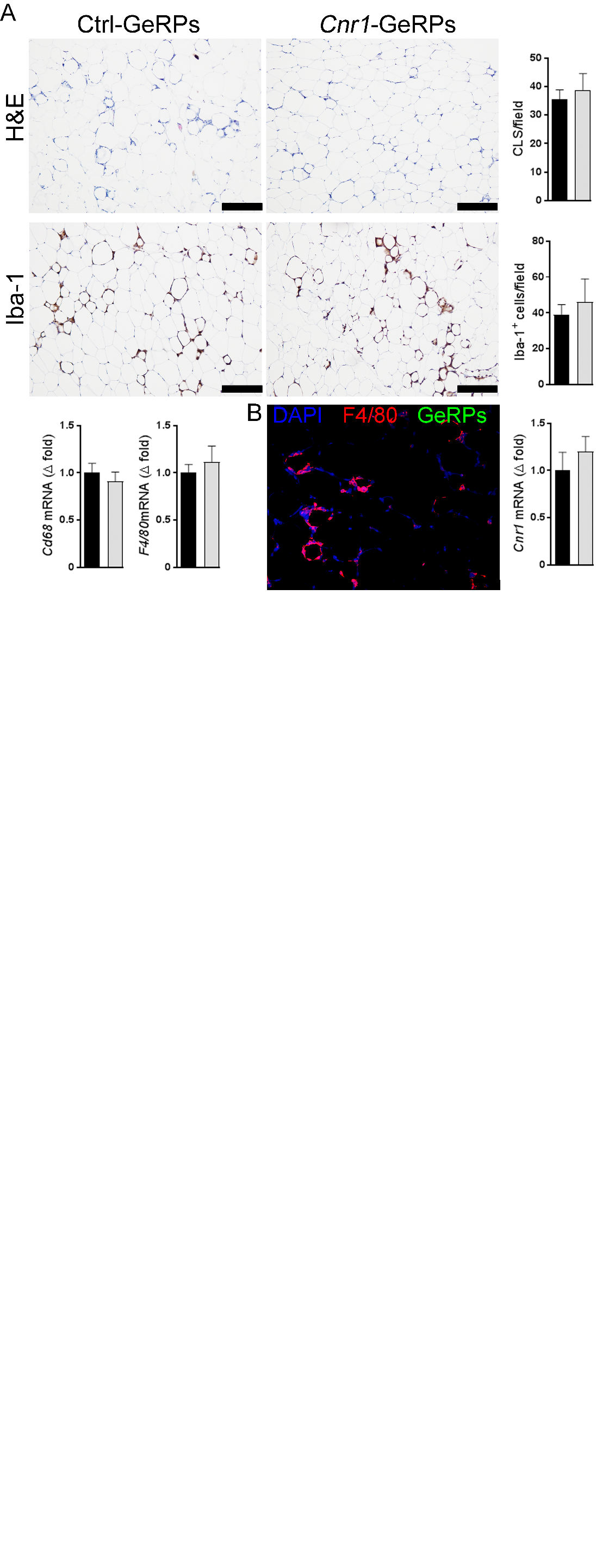


**Supplementary Figure 1: Intravenous-delivery of GeRPs does not influence macrophage content or *Cnr1* expression in periepididymal adipose tissue.**

**A** Adipose tissue morphology and crow-like structure (CLS) quantification from histological slides stained by hematoxylin-Eosin (H&E). Macrophage histology as assessed by Iba-1 immuno-staining (scale bars: 200µm) and whole-adipose tissue gene expression for the macrophages markers *Cd68* and *F4/80*. **B** Representative KCs staining (red) in adipose tissue section from an obese mouse 24 h after intravenous injection with FITC-labeled GeRPs (green) (magnification x200). *Cnr1* gene expression in adipose tissue from DIO mice treated with control (black columns, n=10) or *Cnr1*-GeRPs (light grey columns, n=10). Columns and bars represent means ± SEM. Significant differences from values in control-GeRPs treated obese mice *P<0.05, **P<0.01, *** P<0.001.
